# WHO’s QualityRights Initiative: Transforming Services and Promoting Rights in Mental Health

**Published:** 2020-06

**Authors:** Michelle Funk, Natalie Drew Bold

**Affiliations:** Head of the Policy, Law and Human Rights Unit, Department of Mental Health and Substance Use, World Health Organization, Geneva, Switzerland.; Technical Officer in the Policy, Law and Human Rights Unit, Department of Mental Health and Substance Use, World Health Organization, Geneva, Switzerland.

This perspective essay introduces the World Health Organization’s QualityRights initiative, which uses a multicomponent framework and strategies to promote mental health systems, services, and practices that prioritize respect for human rights, in line with the United Nations Convention on the Rights of Persons with Disabilities (CRPD).^1^ It describes how the initiative is working to provide practical solutions to promote inclusion, legal capacity, and non-coercive approaches in mental health.

Since the adoption of the CRPD in 2006, there has been considerable dialogue, debate, and concerns expressed around the applicability of the Committee on the Rights of Persons with Disabilities’ interpretations of certain key provisions of the convention as they relate to the area of mental health. In particular, these concerns refer to the right to equal recognition before the law and to legal capacity (article 12), as well as the right to liberty and security of the person (article 14).^2^ They are reflected in the declarations and reservations submitted by several countries in relation to these articles specifically and briefly summarized below.^3^

Article 12 of the convention states that all persons with disabilities, including persons with mental health conditions and psychosocial disabilities, must be allowed to exercise legal capacity—in other words, to make decisions for themselves on all aspects of their lives—on an equal basis with others.^4^ This challenges and ultimately prohibits practices such as forced admission and treatment, guardianship, and other forms of substitute decision-making. Instead, article 12 recognizes and promotes the concept of “supported decision-making,” wherein people are provided with access to a variety of support options, including the support of people they trust (for example, family, friends, peers, advocates, lawyers, and personal ombudspersons), in order to make decisions and choices for themselves. This approach recognizes that building on people’s unique abilities and providing them with the support they require allows them to make their own decisions. A person may need support to understand the information, weigh different options, understand the possible consequences of different options, and communicate their decisions to others.^5^

Article 14 on the right to liberty and security of the person states that persons with disabilities should not be deprived of their liberty unlawfully or arbitrarily and the existence of a disability shall in no case justify detention.^6^ Applied in the mental health context, this means that persons with mental health conditions and psychosocial disabilities cannot be involuntarily detained in mental health services or other facilities such as institutions, prayer camps, sheds, or houses. Furthermore, detention on the basis of a diagnosed or perceived disability is not allowed, even when additional reasons or criteria are given for the detention, such as “need for treatment,” “presumed danger,” or “lack of insight.”^7^

The main point of dispute for many professionals in the mental health field and beyond is that these provisions, in the context of “no exceptions,” risk undermining the rights to the enjoyment of the highest attainable standard of health, to access to justice, to liberty, and to life.^8^ They argue that “exceptional” measures such as guardianship and involuntary admission and treatment are necessary to prevent danger to one’s self or others and to ensure that people receive the care and support they need.^9^

For example, Melvyn Freeman et al. argue that prohibiting involuntary admission or treatment “closes viable options for saving lives and is especially tragic where the suicidal ideation is directly linked with impaired decision-making capacity and could have been changed through admission or treatment.”^10^ Similarly, the authors go on to state that “in rare instances others might be harmed or their lives taken in select circumstances, whereas admission or treatment of a person with mental disability might prevent this” and that “when there is a conflict between different rights, the right to life should trump other rights.”^11^

Paul Appelbaum, in a 2019 article, expresses concern that, under the CRPD, older persons with dementia who may be unable to care for their own needs or finances cannot be compelled to have a guardian manage different aspects of their lives. He also deplores the fact that people with major depression or who are experiencing psychosis and refuse to eat cannot be compelled to go to a hospital and that someone in “manic stage of bipolar disorder may be free to spend family savings or wreck the family business.”^12^ He further argues that “[i]n the name of protecting all these people from discrimination, they would be free to destroy their own lives and ruin the lives of their loved ones.”^13^

Many other diverse groups—including mental health professionals, people with lived experience, people with psychosocial disabilities, lawyers, human rights advocates, and other stakeholders—point out that it is the “exceptions” in laws around involuntary admission, treatment, seclusion, and restraint and other coercive measures that, in reality, go on to become standard practice in countries everywhere.^14^ Many call for the complete prohibition of all coercive practices, citing the absence of evidence of these practices’ effectiveness and the available evidence demonstrating that coercive practices cause harm to physical and mental health, can lead to death, can undermine trust within therapeutic relationships, and are incompatible with the fundamental principles of dignity and autonomy.^15^

In an open letter to the World Psychiatric Association in relation to volume 18/1 of *World Psychiatry*, a consortium of users and survivors of psychiatry state that “the option of forced psychiatric treatment affects each citizen (although not equally) and has implications for anyone who comes into contact with the mental health system. The related debate can therefore not continue based on ‘exceptional’ cases and constructed scenarios only.”^16^

For many, the focus now needs to center on putting in place creative responses in mental health care that respect people’s will and preference and foster therapeutic relationships based on trust and empowerment, and in ways that avoid the pitfalls of the past.^17^ In reality, however, laws and standard practice in all countries continue to adopt an “exceptions” approach and to authorize the use of coercive measures, including substitute decision-making, forced detention and treatment, and seclusion and restraint. With polarized opinions on these issues, resistance to adopting alternatives to coercive practices, and entrenched systems, mechanisms, and legal frameworks that keep them in place, mental health reform remains extremely challenging in all countries.

## Fostering dialogue and action toward implementation of the CRPD

An important goal of the WHO QualityRights initiative is to provide practical solutions to promote human rights throughout countries’ mental health and social care systems and in particular to support countries—the vast majority of whom have ratified the CRPD—to actualize the rights of convention.

The initial emphasis of QualityRights was on promoting sustainable change in attitudes and practices in the field of mental health and to ensure respect for the human rights of people with mental health conditions and psychosocial disabilities. Answering questions such as “How should practitioners be practicing in services in order to be compliant with the CRPD?” and “How should practitioners, families, and others respond to crisis situations in a way that respects the legal capacity and right to liberty of the person concerned?” became paramount in these efforts.

Following research on good practices in the literature and an extensive process of consultation with a wide variety of international experts on the types of practices on the ground that align with the CRPD, the next step was to develop strategies to integrate these practices into countries’ mental health systems and services. This involved the development of three major areas of work, which are at the core of the WHO QualityRights initiative:

- building capacity among all stakeholders to improve attitudes and practices to address stigma and discrimination and promote human rights and recovery
- supporting countries in the creation of community-based services and supports that respect and promote human rights
- supporting national policy and law reform in line with the CRPD and other international human rights standards

Key to the initiative is the active engagement and support of civil society, in particular organizations of persons with lived experience, in all these areas of the work.

## Strengthening knowledge and capacity on rights and recovery

On November 27, 2019, WHO launched its QualityRights training and guidance materials and tools.^18^ These resources aim to support countries in transforming their health systems and services toward a person-centered, recovery-oriented, and human rights-based approach in line with the CRPD and the vision outlined by WHO Director General Tedros Ghebreyesus in the foreword to the QualityRights materials.

In order to maximize reach, WHO has also developed the QualityRights e-training on mental health, disability, human rights, and recovery. This platform has the potential to reach tens of thousands of stakeholders within and across countries.

The QualityRights resources are designed to build capacity on mental health, disability, human rights, and recovery among a full range of stakeholders, including policy makers, health and mental health professionals, social workers, people with lived experience, organizations of persons with disabilities, families and care partners, nongovernmental organizations, professional associations and organizations, academic institutions, and other key national actors.

The tensions, difficulties, and challenges highlighted earlier around involuntary admission and treatment, seclusion and restraint, and other coercive practices are addressed head on in the content of the QualityRights training modules. The modules build knowledge and skills on concrete strategies for promoting treatment, care, and support based on people’s will and preference, even in the most challenging of circumstances. This includes a variety of strategies for promoting legal capacity, including supported decision-making, advance planning, best interpretation of will and preference, and Ulysses clauses.^19^ The modules also build knowledge and skills in order to avoid coercion. They outline how to develop and use individualized plans that address a person’s sensitivities and specific situations that can lead to distress and agitation. Furthermore, the modules provide techniques for de-escalating and resolving conflicts, creating a “saying yes” and “can do” culture, establishing supportive environments and comfort rooms, and setting up response teams to manage challenging and conflictual scenarios.^20^

Of course, conflictual situations are sometimes unavoidable, and even with the best of measures and strategies in place that align with the rights of the CRPD, coercive practices may occur on occasion. However, in these situations the QualityRights materials also provide guidance and training on how to learn from such incidents, understand what went wrong, and undertake measures to prevent them from recurring in the future.

A key ingredient to the effectiveness of the QualityRights materials and tools in changing attitudes and practices lies in their design and methodology. The materials use exercises, debates, discussions, case studies, and scenarios from countries around the world to engage people, on a personal and emotional level, with the concepts of human rights and recovery. People are encouraged to explore what rights and recovery means to them personally, in order to gain a more profound understanding of their importance to others, notably people with lived experience.

Through exercises and case scenarios, for example, people explore what it might be like to be denied the right to make decisions about all aspects of their lives—what to eat, what to wear, where to live, what kind of treatment to receive, how to handle personal and financial matters, and so on—as is the experience of so many people with psychosocial disabilities. In order to better understand concepts underpinning recovery, trainees are also encouraged to think about and discuss what has helped them recover from situations or events in their own lives—friendship, support, having hope, and finding purpose and meaning in life—in order to better understand that these are important for everyone in recovery, including people with psychosocial disabilities.

The face-to-face QualityRights training and guidance materials in their pilot form, as well as the QualityRights e-training platform, have already been extensively implemented in countries in all regions.

## Support for transforming mental health and related services

Another area of work being undertaken as part of QualityRights is to support countries in promoting community-based and recovery-oriented mental health and related services that respect and promote human rights.

The WHO QualityRights assessment toolkit enables countries to assess their services against standards derived from the CRPD.^21^ Furthermore, the recently published module on transforming services and promoting human rights also provides countries with the framework, guidance, and training required to address gaps and transform services in line with CRPD standards.^22^ Some of the issues addressed in this “transformation” guidance tool include changing the service culture and the power dynamics, defining a shared vision for the service, and working on the specific priorities and actions for change.

In addition, as part of this area of work, the QualityRights initiative is developing a document that will showcase community-based mental health services being implemented in countries around the world that respect human rights, are person-centered and recovery-oriented, operate without coercion, and promote autonomy, participation, and inclusion in line with the CRPD. The document will encompass mental health services from all regions of the world, reflecting different socioeconomic and cultural contexts. The guidance document will discuss the applicability of services in different settings and underscore the importance of not importing models inappropriately into different contexts. Showing that these types of services exist and are effective is critical key to inspiring policy makers and other actors to spread these new and innovative approaches to mental health across the world.

### Aligning policy and law with the CRPD

The third area of work that WHO has embarked on as part of QualityRights is the development of new guidance for countries on how to formulate and implement mental health-related policy and law in line with the CRPD.

Many countries rely on assistance and support from WHO to develop or reform their national laws and policies related to mental health. Previous WHO guidance in these areas were drafted prior to the coming into force of the CRPD and thus does not comply fully with the standards set by the convention.

Indeed, the lack of clear and concrete guidance on policy and law in the new CRPD era remains a major barrier to countries seeking to ensure that their mental health laws and policies comply with human rights standards. Policy makers are required to go beyond simply repealing provisions related to forced admission, treatment, and guardianship. They need clear legal and policy directions that provide practical solutions and strategies for upholding the rights of people with psychosocial disabilities and for ending coercion and abuse in mental health.

WHO’s new policy and law guidance will answer critical questions such as “How can we safeguard people’s rights, even in crisis situations?”; “What are the processes for respecting peoples will and preferences?”; “What concrete measures are needed to establish supported decision-making processes?”; and “How can policy and law facilitate the development of community-based services that promote recovery and rights?”

Creating strong policy and legislative frameworks that align with the CRPD will be critical to ensuring that practices and services on the ground respect and promote rights and recovery for people with psychosocial disabilities effectively and sustainably.

## Country support

Implementation of the WHO QualityRights initiative started several years ago with small pilot projects in different countries. From 2014 to 2016, a comprehensive statewide implementation of QualityRights was undertaken in the state of Gujarat in India, led by the Ministry of Health and Family Welfare. As part of the study project, assessments of quality and human rights conditions were conducted in services throughout the state, and individualized improvement plans were developed at each of the services using the QualityRights tools and methodology highlighted above. Additionally, a comprehensive capacity-building program was undertaken to train health care staff, people using services, and their families using the QualityRights training modules.

The project evaluation demonstrated significant changes over the course of the three years, including substantial improvement in the quality of care, attitudes toward people using services, and satisfaction and empowerment among people using services.^23^

Since the initiative in Gujarat, QualityRights continues to gain momentum in countries in all regions of the world. During 2019, nationwide launches and rollouts of QualityRights were initiated in Ghana, the Philippines, Kenya, Turkey, Estonia, and Czechia; more launches are scheduled for 2020. QualityRights activities are also continuing on a wide scale in Lebanon, Armenia, Bosnia and Herzegovina, Romania, Slovakia, Croatia, and Lithuania. Although activities vary from country to country, activities include QualityRights service assessments, the implementation of transformation plans, face-to-face capacity building, the rollout of e-training, and policy and law reform. The actions taken to date and achievements are detailed country by country on the WHO QualityRights Country Implementation Portal.

This portal has been created to enable countries to document their activities and share information, strategies, experiences, and resources.^24^ Documenting QualityRights reform in countries, and showing that real and impactful change is possible, will be key to inspiring other countries to take on the challenge and commitment of promoting human rights and recovery in mental health.

Although positive results are being achieved, there remain challenges in implementation and sustainability at the individual, service, and systems levels. At an individual level, not all staff are convinced of the need to change practices, and misconceptions and discriminatory attitudes persist. More time is required to reinforce new knowledge and skills in order to facilitate more sustained attitude change among larger numbers of staff working in mental health services, and this also needs to be reinforced by attitudinal change within the wider community.

Another key challenge concerns the types of services that are offered and the need to have all services be based in the community. In many of the countries engaging in the QualityRights initiative, psychiatric institutions and facilities remain the core of the services being provided. Facilities and institutions that are isolated from the community are breeding grounds for coercive practices, violence, and abuse and therefore need to be phased out. Furthermore, many services are under-resourced both financially and in terms of staff, which is an additional barrier to providing quality care and support in line with human rights standards. And finally, laws that legitimize coercive practices (even under exceptional circumstances only) will always remain a barrier to the full integration of a CRPD approach in mental health.

## Conclusion

Mental health systems in countries around the world are far from reaching the obligations set out in international human rights conventions, in particular the CRPD. There are, however, a number of practical solutions, resources, and tools now available through the WHO QualityRights initiative that have been used in countries and have demonstrated that change is possible and can lead to better outcomes for people using services, professionals, policy makers, and communities. The new QualityRights tools that are currently being developed around good practice community-based services and on CRPD-aligned policy and law will also provide countries with much-needed guidance to achieve sustainable change.

Not all countries are ready or have the capacity to immediately take the multifaceted measures required to align their services and systems with the CRPD. A key motivating factor for many will be the availability of clear, documented evidence of good outcomes from other countries. This will demonstrate that change is possible and will hopefully convince them to embark on similar reform efforts. It will also be crucial for ensuring better research investment around the implementation of the CRPD.

Box 1 Links and resources

- QualityRights materials and tools: https://www.who.int/publications-detail/who-qualityrights-guidance-and-training-tools
- QualityRights self-help recovery tool for mental health and well-being: https://www.who.int/publications-detail/who-qualityrights-self-help-tool
- QualityRights Country Implementation Portal: https://qualityrights.org/
- WHO’s feature story on WHO QualityRights: https://www.who.int/news-room/feature-stories/detail/mental-health-services-in-lebanon-an-approach-focused-on-recovery
- *QualityRights in Lebanon: A personal perspective* (video): https://qualityrights.org/wp-content/uploads/WHO-Mental-Health.mp4

## References

[1] Convention on the Rights of Persons with Disabilities, G.A. Res. 61/106 (2006).

[2] Freeman M. C., Kolappa K., Caldas de Almeida J. M. (2015). “Reversing hard won victories in the name of human rights: A critique of the General Comment on Article 12 of the UN Convention on the Rights of Persons with Disabilities,”. Lancet Psychiatry.

[3] Convention on the Rights of Persons with Disabilities: Declarations and reservations.

[4] Committee on the Rights of Persons with Disabilities, General Comment No. 1, Article 12: Equal Recognition before the Law, UN Doc. CRPD/C/GC/1 (2014).

[5] World Health Organization (2019). Legal capacity and the right to decide: WHO QualityRights Core training; Mental health and social services.

[6] Committee on the Rights of Persons with Disabilities, Guidelines on Article 14 of the Convention on the Rights of Persons with Disabilities, UN Doc. A/72/55 (2015).

[7] World Health Organization (see note 5).

[8] Freeman et al. (see note 2).

[9] Ibid.

[10] Ibid.

[11] Ibid.

[12] Applebaum. P. S. (2019). “Saving the UN Convention on the Rights of Persons with Disabilities—from itself,”. World Psychiatry.

[13] Ibid.

[14] M. Funk, Drew. N. (2019). “Practical strategies to end coercive practices in mental health services,”. World Psychiatry.

[15] Substance Abuse and Mental Health Services Administration (2011). The business case for preventing and reducing restraint and seclusion use.

[16] (2019). Open Letter to WPA.

[17] Puras D., Gooding P. (2019). “Mental health and human rights in the 21st century,”. World Psychiatry.

[18] World Health Organization (2019). QualityRights materials for training, guidance and transformation.

[19] World Health Organization (2019). Supported decision-making and advance planning: WHO QualityRights specialized training.

[20] World Health Organization (2019). Strategies to end seclusion and restraint: WHO QualityRights specialized training.

[21] World Health Organization (2012). WHO QualityRights tool kit to assess and improve quality and human rights in mental health and social care facilities.

[22] World Health Organization (2019). Transforming services and promoting human rights: WHO QualityRights training and guidance: Mental health and social services.

[23] Pathare S., Funk M., Drew N. (2019). “Systematic evaluation of the QualityRights programme in public mental health facilities in Gujarat, India,”. British Journal of Psychiatry.

[24] World Health Organization WHO QualityRights country implementation portal.

